# MedNeXt-Based Automated Tumor Segmentation and TMTV Prognostication in Hodgkin Lymphoma PET Images: A Retrospective Study

**DOI:** 10.3390/cancers18193196

**Published:** 2026-10-03

**Authors:** Haoan Zhang, Fuming Sun, Hang Yu, Xinyuan Chen, Yixuan Shi, Yue Teng, Wenze He, Yu Jin, Shangdong Liu, Chongyang Ding, Chong Jiang, Jingyan Xu

**Affiliations:** 1 Department of Hematology, Nanjing Drum Tower Hospital, School of Basic Medicine and Clinical Pharmacy, China Pharmaceutical University, Nanjing 210008, China; 18606351919@163.com (H.Z.); 3326090006@stu.cpu.edu.cn (F.S.); 3325092363@stu.cpu.edu.cn (W.H.); 18255098126@163.com (Y.J.); 2 School of Computer Science, Nanjing University of Posts and Telecommunications, Nanjing 210008, China; 1224045925@njupt.edu.cn (H.Y.); 1224045737@njupt.edu.cn (Y.S.); lsd@njupt.edu.cn (S.L.); 3 Department of Hematology, Nanjing Drum Tower Hospital, Gulou Clinical Medical College, Nanjing University of Chinese Medicine, Nanjing 210008, China; 18384537698@163.com; 4 Department of Nuclear Medicine, Nanjing Drum Tower Hospital, Medical School of Nanjing University, Nanjing 210008, China; 18304636833@163.com; 5 Department of Nuclear Medicine, Jiangsu Provincial Hospital, Nanjing Medical University, Nanjing 210029, China; chongyangding@163.com; 6 Department of Nuclear Medicine, West China Hospital, Sichuan University, Chengdu 610041, China

**Keywords:** Hodgkin lymphoma, metabolic tumor volume, MedNeXt, segmentation, PET

## Abstract

**Background/Objectives**: To evaluate a deep learning–based method for automatic segmentation of Hodgkin lymphoma (HL) on PET images and to explore the prognostic value of total metabolic tumor volume (TMTV) derived from segmentation. **Methods**: PET datasets from two independent institutions were retrospectively collected and divided into training and validation cohorts. MedNeXt was trained for HL PET segmentation and benchmarked against five representative segmentation architectures under the same data split, preprocessing pipeline, and evaluation framework. Segmentation performance was assessed using the Dice similarity coefficient (DSC), Jaccard index (JSC), 95th percentile Hausdorff distance (HD95), and average symmetric surface distance (ASSD). Bland–Altman and linear regression analyses evaluated agreement between ground-truth TMTV (gtTMTV) and predicted TMTV (pTMTV). Cox proportional hazards regression analysis was applied to assess the prognostic value of pTMTV. **Results**: In the validation cohort, the mean values (±standard deviation) of DSC, HD95, JSC, and ASSD were 0.702 ± 0.191, 67.998 ± 99.220, 0.561 ± 0.203, and 16.992 ± 31.659, respectively. Among the evaluated segmentation models, MedNeXt showed the best segmentation performance. Mean gtTMTV and pTMTV were 424.681 ± 498.811 cm^3^ and 405.871 ± 466.984 cm^3^, showing good agreement. pTMTV was significantly associated with progression-free survival (PFS) (*p* = 0.048), but not overall survival (OS) (*p* = 0.163). **Conclusions**: MedNeXt achieved reliable HL segmentation, although tumor boundary delineation requires further improvement. The derived pTMTV may serve as a prognostic indicator for PFS but showed no significant association with OS. Further validation in larger cohorts is warranted.

## Simple Summary

Hodgkin’s lymphoma (HL) is a blood cancer that mainly affects young adults and older individuals. Although most patients are cured, some experience treatment failure or relapse, making it important to identify high-risk patients. The total metabolic tumor volume (TMTV) on PET/CT scans is a useful prognostic indicator, but manual measurement is time-consuming and operator-dependent. In this study, we used a deep learning model (MedNeXt) to automatically segment HL lesions on PET images from 192 patients across two centers. The model achieved moderate-to-high accuracy (DSC = 0.702), and the predicted TMTV was potentially associated with progression-free survival (*p* = 0.048), though not with overall survival (*p* = 0.163). These findings suggest that predicted TMTV has the potential to serve as a practical tool for standardized risk stratification in HL, pending further validation in larger cohorts.

## 1. Introduction

Hodgkin lymphoma (HL) is a malignant tumor of the lymphatic system that mainly affects adolescents, young adults, and older individuals [1,2]. Although HL has relatively high cure rates, 5–10% of patients do not achieve remission after the initial treatment, and 10–30% of those who do experience relapse or disease progression [1,2]. This highlights the critical importance of comprehensive initial evaluation and prognostic risk stratification for HL patients. 18F-FDG PET/CT plays a crucial role in various aspects of HL management, including disease diagnosis, treatment response monitoring, and predicting survival outcomes [1,2,3]. Semi-quantitative PET parameters, such as total metabolic tumor volume (TMTV) and total lesion glycolysis (TLG), have been validated as independent prognostic biomarkers for HL patients [4]. Accurate segmentation of tumor lesions is essential for obtaining reliable quantitative metabolic parameters. However, current manual segmentation methods are limited by inherent factors such as subjectivity and time consumption, which hinder the clinical use of PET-derived quantitative parameters. This ongoing challenge highlights the urgent need to develop standardized automated segmentation protocols.

Convolutional neural network (CNN)-based architectures are predominantly used in medical image segmentation [5]. Cairns et al. [6] showed that an automated segmentation tool using a 3D U-Net architecture could improve the quantification of metrics like TMTV and TLG. Hu et al. [7] advanced the field by integrating multi-view perspectives with 3D networks, showing strong potential for lymphoma segmentation. However, these approaches mainly use CNNs in localized modules, limiting further improvements in segmentation performance.

This study uses the MedNeXt architecture, a fully 3D convolutional neural network designed for medical image segmentation. The framework preserves the inductive biases of CNNs and integrates large convolutional kernels with residual connections. This design improves long-range dependency modeling in medical images, enhances feature preservation during downsampling/upsampling, and boosts gradient propagation efficiency, enabling strong performance even in data-constrained conditions. Experimental studies [5,8] show that this configuration improves the segmentation accuracy for complex anatomical structures (e.g., organ boundaries), captures semantic features, and maintains training stability with limited data. Moradi et al. [9] confirmed MedNeXt’s superior performance in segmenting gross tumor volume of primary and metastatic lymph nodes. Kanakarajan et al. [10] reported excellent segmentation efficacy for brain metastases on MRI (DSC: 0.94 ± 0.03), while Rosa et al. [11] showed that the MedNeXt-inspired BrainAgeNeXt model predicted brain age with high accuracy (MAE: 2.78 ± 3.64). However, current MedNeXt-based segmentation models have critical limitations, including poor generalizability across tumor subtypes and limited clinical validation in HL contexts, requiring rigorous multi-center studies to verify their clinical utility.

In this study, a deep learning model based on the MedNeXt architecture was used to automatically segment lymphoma lesions in PET images of HL patients, aiming to evaluate segmentation performance and validate its prognostic value.

## 2. Materials and Methods

### 2.1. Study Datasets

A total of 192 patients with pathologically confirmed HL from January 2014 to May 2024 were retrospectively included from two institutions: Jiangsu Provincial People’s Hospital and Nanjing Drum Tower Hospital. The cohort comprised 114 males and 78 females, with a median age of 34 years (range, 12–83 years). Patients were divided into training and validation cohorts based on the institution: cases from Jiangsu Provincial People’s Hospital (*n* = 114) were assigned to the training cohort, while cases from Nanjing Drum Tower Hospital (*n* = 78) constituted the independent external validation cohort. The inclusion criteria for this study were as follows: (1) confirmation of histopathology consistent with HL; (2) a positron emission tomography/computed tomography (PET/CT) scan before treatment; (3) administration of the standard chemotherapy regimen (doxorubicin, bleomycin, vincristine, and dacarbazine, (ABVD); (4) complete clinicopathologic and follow-up data. The exclusion criteria were as follows: (1) patients who had previously undergone antitumor therapy; (2) patients with a history of other tumors; (3) patients with incomplete clinical or imaging data; (4) patients with other malignancies or serious diseases. This study was approved by the Ethics Committees of Nanjing Gulou Hospital (Approval No. 2024-397-01) and Jiangsu Provincial People’s Hospital (Approval No. 2015-NT-001). Subjects were exempted from signing the informed consent form.

### 2.2. Image Acquisition

The acquisition protocol is as follows. All patients were required to fast for at least six hours before undergoing molecular imaging. This was to ensure that the blood glucose concentration did not exceed 11.1 mmol/L during fasting. Following the principle of individualized drug administration, 18F-FDG (Nanjing Jiangyuan Andike Positron and Development Co., Ltd., Nanjing, China, radiological purity > 95%) was administered through a superficial vein in the upper limbs. The dose was controlled between 3.70 and 5.55 MBq/kg body weight. After the radiotracer injection, patients were required to rest for 50 to 60 min to allow biodistribution equilibrium. Image acquisition was performed using a Siemens Biograph 16 HR PET/CT scanner (Siemens Healthineers, Erlangen, Germany) for the 117 patients from Jiangsu Province Hospital, and a Philips GXL-16 PET/CT scanner (Philips Healthcare, Amsterdam, The Netherlands) for the 78 patients from Nanjing Drum Tower Hospital. The patient was placed in the standard supine position, and the axial scan covered from the base of the skull to the mid-femur, extending to the plantar region if clinically indicated. The CT subsystem used a 120 kV tube voltage and 100 mA tube current, with a soft tissue convolution kernel for image reconstruction and a layer thickness of 2 mm. The imaging procedure strictly followed the “CT first-PET second” protocol, using 3D acquisition mode to acquire images from a single bed. The acquisition time for the single bed was 2–3 min. PET images were reconstructed using an ordered subset maximum expectation value algorithm after attenuation correction based on CT scan data. According to the final enrollment list, baseline PET/CT DICOM files for each patient were exported from the imaging report system.

### 2.3. Ground Truth Generation

The quantitative analysis of imaging parameters was conducted using the LIFEx software (version 7.3.0) medical image processing platform [12,13]. A semi-quantitative segmentation algorithm was applied to the 18F-FDG PET/CT image data for lesion segmentation. The threshold for this algorithm was set at 41% of SUVmax [3,14]. The system automatically calculated the following metabolic parameters: maximum standardized uptake value (SUVmax), total metabolic tumor volume (MTV), and total lesion glycolysis (TLG). TMTV was defined as the sum of the MTVs of all malignant foci, while TLG was calculated by multiplying TMTV and the SUVmean of the corresponding region. Anatomical structures with basal metabolic activity include the central nervous system, urinary and excretory systems, and digestive system, among others. It was necessary to exclude these elements from the control during image processing. Manual correction of ROI boundaries based on CT anatomical localization was performed when spatial overlap occurred between lesions and physiological hypermetabolic regions. Abnormal metabolic foci in the hematopoietic system and hepatic parenchyma (regional SUVmax ≥ 2.5 or focal SUVmax > 1.5 times the liver background) should be included in the pathological metabolic volume calculation [13]. To ensure data reliability, The annotation process was conducted by two board-certified nuclear medicine physicians with 5 years of experience. Prior to the delineation, a consensus meeting was held to establish standardized annotation guidelines. Subsequently, the two physicians independently reviewed and segmented the lesions. In cases of disagreement, a senior nuclear medicine physician with over 10 years of experience made the final decision.

### 2.4. Image Preprocessing

This study included only PET images derived from PET/CT scans. To ensure quantitative consistency across different scanners, PET image reconstruction was harmonized following the EARL2 standards. All PET images were converted to standardized uptake values (SUVs) to ensure biological comparability across subjects. To address variations in acquisition protocols, all images were resampled to an isotropic resolution of 2.0 × 2.0 × 2.0 mm^3^ using trilinear interpolation. The corresponding ground-truth masks were resampled using nearest-neighbor interpolation. Intensity normalization was performed using a foreground-based Z-score method. Specifically, the mean and standard deviation of non-zero voxels in the training set were calculated, and intensities were normalized to zero mean and unit variance. To preserve high-frequency features essential for small lesion detection, no additional Gaussian smoothing or denoising was applied. During training, data augmentation techniques, including rotation, translation, and scaling, were applied to increase data diversity and reduce overfitting. To address class imbalance in medical images, where tumor regions typically occupy a small proportion, a composite loss function was designed. Specifically, weighted cross-entropy and Dice loss were combined, with weights of 1.7 and 1.0, to optimize voxel-wise classification accuracy and regional overlap.

### 2.5. Model Architecture and Training

The MedNeXt architecture was adopted, comprising four symmetric encoder–decoder stages, each with bottleneck blocks. MedNeXt core computational units were used for both downsampling and upsampling. Deep supervision was applied in the decoder, where lower weights were assigned to losses from deeper, low-resolution feature maps. Additive residual connections were used, and padded convolutions preserved feature map resolution. In addition, MedNeXt incorporates the UpKern initialization strategy, which initializes large-kernel networks from pretrained small-kernel models via trilinear upsampling and progressively increases the kernel size. This approach mitigates performance saturation when training large-kernel networks on limited medical datasets, thereby improving performance on small-scale medical imaging data.

**Figure 1:**
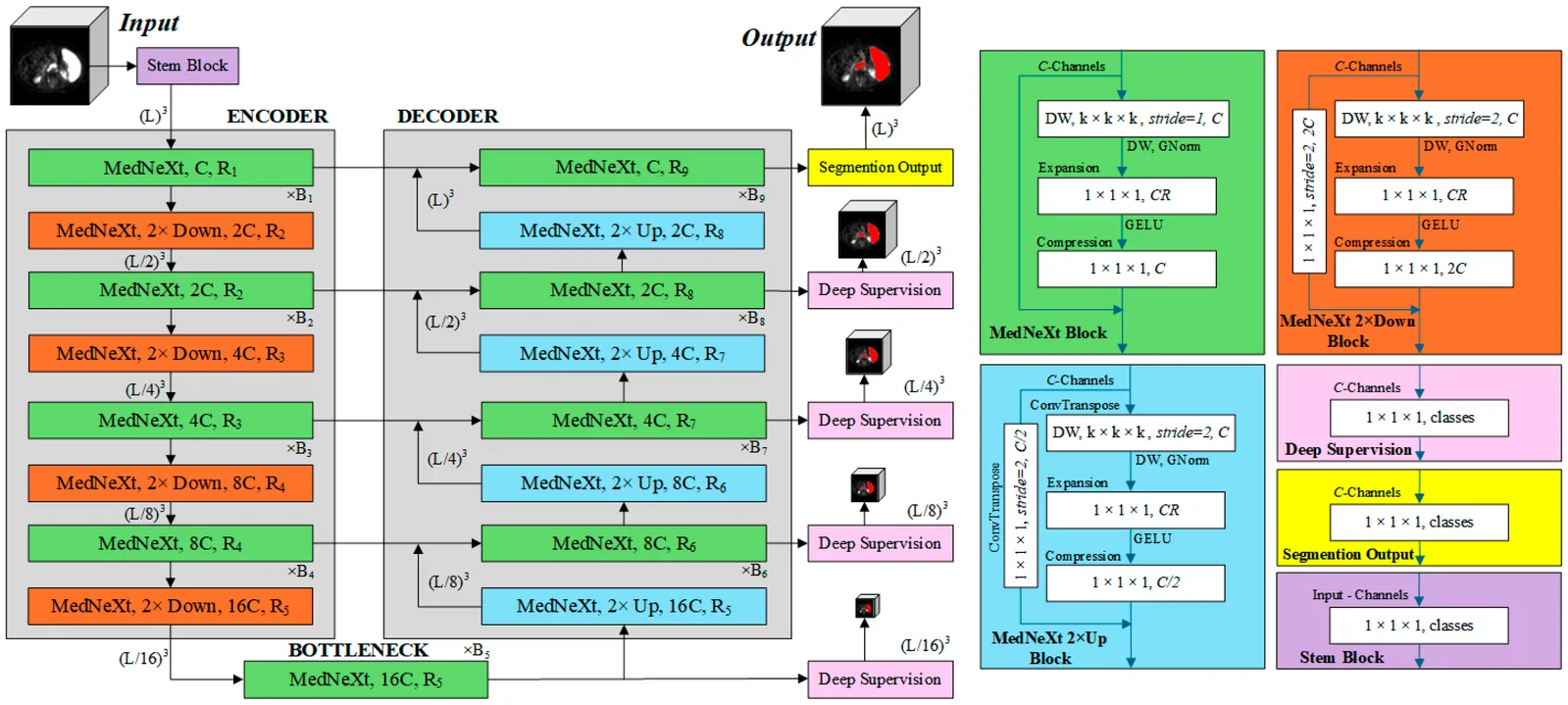
The architecture of MedNeXt.

During training, deeply over-parameterized convolutions were used to enhance 3D spatial feature representation. A kernel size of 3 × 3 × 3 was used to balance computational efficiency and the receptive field. Due to the high memory demands of 3D convolutions, the batch size was set to 2. Input images were cropped into 128 × 128 × 128 patches to meet the model input requirements. A hybrid loss function combining cross-entropy and Dice loss was used, with weights of 1.7 and 1.0, respectively, to address class imbalance. The model was optimized using AdamW, with an initial learning rate of 1 × 10^−3^ and a weight decay of 1 × 10^−5^. A polynomial (“Poly”) learning rate decay strategy was applied, defined as (1 − epoch/max_epoch)^0^·^9^. The network architecture is shown in (Figure 1). The model was trained for 1000 epochs on an NVIDIA 3090 GPU. The final model was selected based on the highest Dice similarity coefficient on the validation cohort.

### 2.6. Segmentation Performance

We computed the Dice similarity coefficient (DSC), false negative rate (FNR), Jaccard index (JSC), false positive rate (FPR), negative predictive value (NPV), positive predictive value (PPV), sensitivity, specificity (SPE), 95th percentile Hausdorff distance (HD95), and average symmetric surface distance (ASSD).

To benchmark MedNeXt, we additionally included five representative segmentation architectures (3D UXNet, nnFormer, nnU-Net, TransBTS, and UNETR). To ensure a fair comparison, all models were trained and evaluated under identical conditions, including the same data split, preprocessing pipeline, data augmentation, optimization strategies, and hyperparameter tuning protocols as MedNeXt. Segmentation performance was assessed using the Dice similarity coefficient (DSC), Jaccard index, positive predictive value (PPV), and sensitivity. As the primary objective of this study was to evaluate the segmentation performance of MedNeXt and the prognostic value of pTMTV derived from MedNeXt-based segmentation, prognostic analyses were performed exclusively on the MedNeXt-derived segmentation results.

### 2.7. Statistical Analysis

Statistical analysis was performed using IBM SPSS 22.0 and R4.2.2 software. The distribution of quantitative data was assessed using the Shapiro–Wilk test (SW). Data not following a normal distribution were expressed as M (Q1, Q3). Qualitative data were expressed as frequency (percentage).

The Bland–Altman method was used to assess the consistency between ground truth TMTV (gtTMTV) and predicted TMTV (pTMTV). The relationship between gtTMTV and pTMTV was evaluated using linear regression analysis. The results were presented as scatter plots with fitted regression lines, and the strength of the association was quantified using the Pearson correlation coefficient. The primary endpoints were progression-free survival (PFS) and overall survival (OS). PFS was defined as the time from initial disease diagnosis to disease progression, recurrence, death from any cause, or the most recent follow-up visit. Survival was evaluated using the Kaplan–Meier method and compared with the log-rank test. OS was defined as the time from initial disease diagnosis to death from any cause or the conclusion of follow-up. Survival outcomes were estimated using the Kaplan–Meier method and compared using the log-rank test. Time-dependent receiver operating characteristic (ROC) curves were used to evaluate the predictive performance of pTMTV at multiple time points. Univariable and multivariable Cox proportional hazards regression analyses were employed to explore the predictive value of potential independent response predictors. Statistical significance was determined by the *p*-value, with a threshold of 0.05 indicating relevance.

## 3. Results

### 3.1. Dataset Characteristics

A total of 192 patients from two institutions were included in this study and divided into a training cohort (*n* = 114) and a validation cohort (*n* = 78) based on institution. The median follow-up duration was 38 months (range: 5–134 months). During follow-up, 60 patients experienced disease recurrence or progression, and 22 died. The demographic and clinical characteristics of these patients are shown in Table 1.

### 3.2. Segmentation Performance in the Validation Cohort

**Figure 2:**
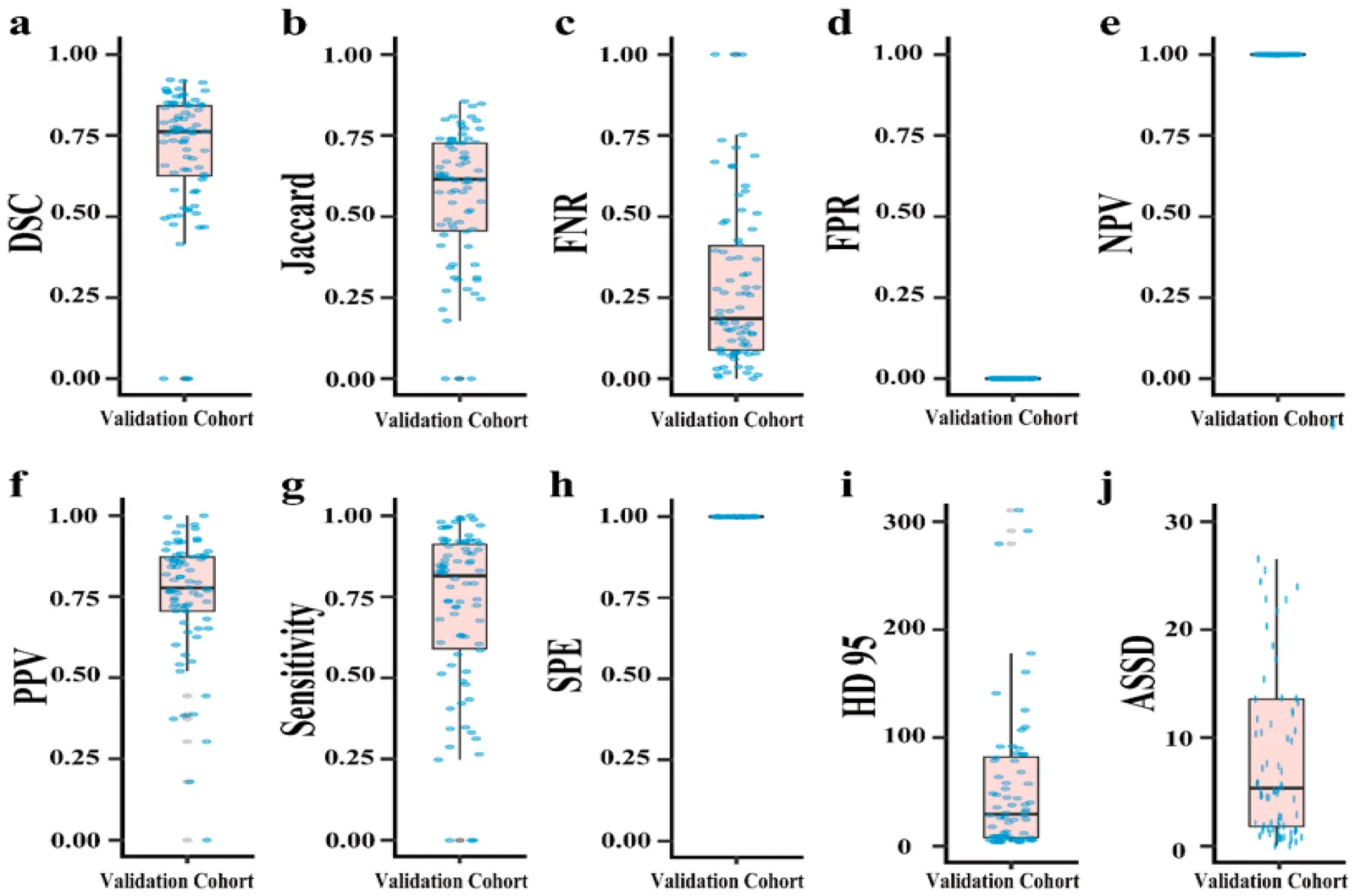
Boxplot plus scatterplot displaying the distribution of the segmentation performance parameters in the validation cohort. (**a**) DSC, (**b**) Jaccard, (**c**) FNR, (**d**) FPR, (**e**) NPV, (**f**) PPV, (**g**) sensitivity, (**h**) SPE, (**i**) HD95, and (**j**) ASSD. Blue dots represent individual values for each case. The pink box indicates the interquartile range (IQR), with the horizontal line denoting the median. The upper and lower whiskers extend to 1.5 × IQR, and gray dots represent outliers beyond the whiskers.

In the validation cohort, the mean values (±standard deviation) of the DSC, FNR, FPR, HD95, Jaccard, NPV, PPV, sensitivity, SPE, and ASSD were 0.702 ± 0.191, 0.283 ± 0.249, 0.00026 ± 0.00036, 67.998 ± 99.220, 0.561 ± 0.203, 0.9996 ± 0.0006, 0.750 ± 0.185, 0.716 ± 0.249, 0.9997 ± 0.0003, and 16.992 ± 31.659, respectively. The statistical analysis is presented in Table 2 and Figure 2.

Different segmentation methods were compared in the validation cohort under the same experimental setting. MedNeXt achieved the best performance, with superior accuracy and robustness in terms of the DSC, Jaccard, PPV, and sensitivity. nnU-Net achieved the second-best performance, with a DSC, Jaccard, PPV, and sensitivity of 0.687 ± 0.199, 0.544 ± 0.204, 0.745 ± 0.192, and 0.721 ± 0.249, respectively. nnFormer and 3D UXNet showed lower segmentation accuracy, with DSC values of 0.668 ± 0.228 and 0.618 ± 0.236, respectively. TransBTS showed moderate performance, with a DSC and Jaccard index of 0.620 ± 0.247 and 0.491 ± 0.236, respectively. In contrast, UNETR showed the poorest performance among all methods, with a DSC, Jaccard, PPV, and sensitivity of 0.485 ± 0.291, 0.371 ± 0.264, 0.512 ± 0.328, and 0.657 ± 0.276, respectively. Detailed results are provided in Table 3. To explore the potential clinical impact of segmentation errors on pTMTV-based risk stratification, we conducted Cox regression analyses to assess whether segmentation performance metrics, including the DSC, HD95, and ASSD, were associated with survival outcomes. No significant associations were observed (*p* = 0.621, 0.986, and 0.989, respectively), indicating that segmentation performance did not appear to materially influence pTMTV-based prognostic stratification in the present cohort. We stratified the validation cohort into groups based on the median values of the DSC, HD95, and ASSD. We found that only the DSC groups showed a significant correlation with pTMTV (*p* = 0.002), whereas the differences in pTMTV across the HD95 and ASSD groups were not significant (*p* = 0.135 and 0.232, respectively). Using a median cutoff of 0.76 for the DSC, the mean pTMTV for the low-DSC group (indicating poorer segmentation performance) was 290.128 ± 348.563 compared to 591.061 ± 576.181 for the high-DSC group (indicating better segmentation performance). These findings suggest a certain correlation between segmentation performance and pTMTV magnitude; specifically, higher pTMTV values were associated with higher DSC and thus better segmentation performance. Conversely, patients with lower pTMTV exhibited lower DSC values, indicating poorer segmentation performance. This, to some extent, reflects that there is still room for improvement in the model’s ability to identify and segment small lesions.

### 3.3. Predicted TMTV in Validation Cohort

**Figure 3:**
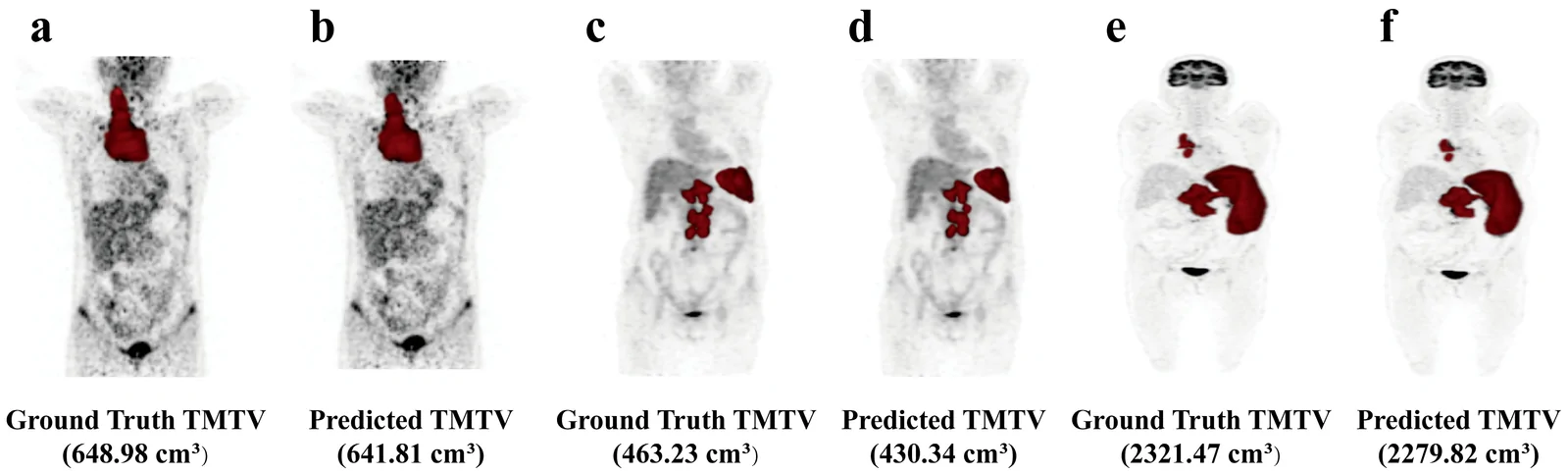
Examples of ground truth (**a**,**c**,**e**) and predicted (**b**,**d**,**f**) total metabolic tumor volume (TMTV) masks, with the red color superimposed over the PET maximum intensity projection.

**Figure 4:**
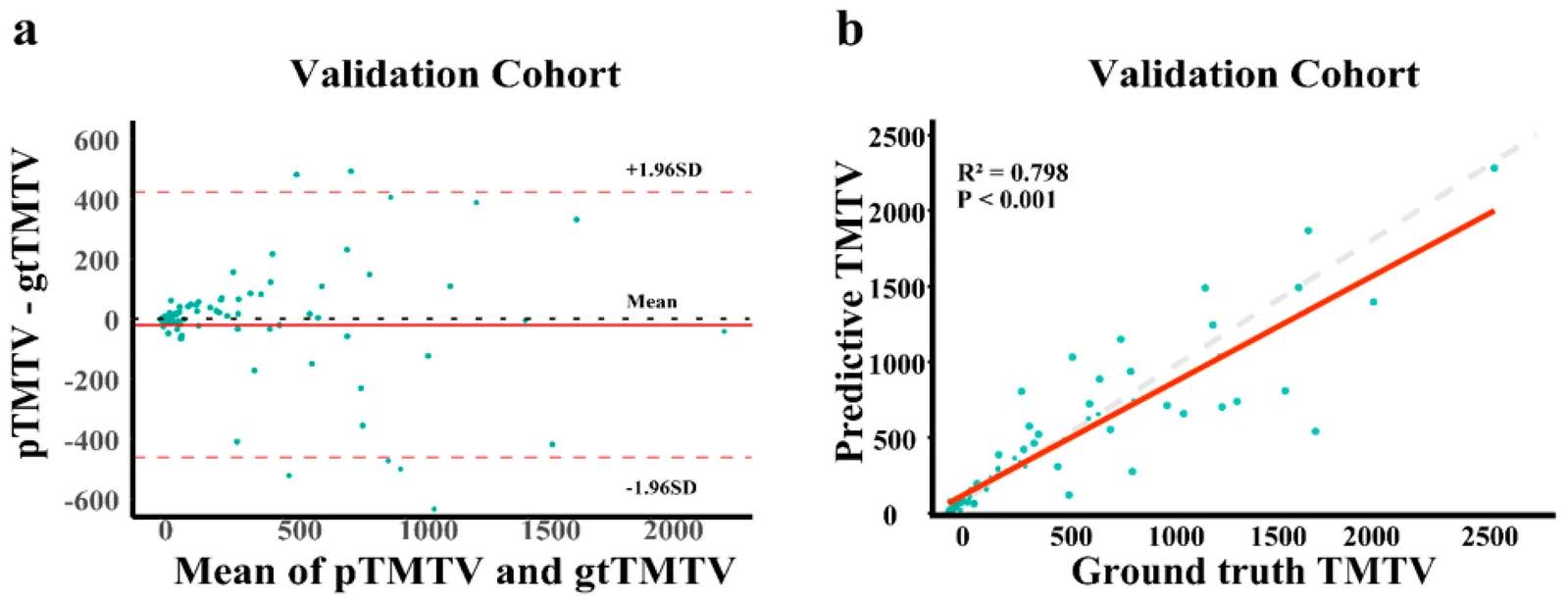
Bland–Altman plot comparing gtTMTV and pTMTV in the validation cohort (**a**). Regression analyses between gtTMTV and pTMTV in the validation cohort (**b**). The dashed gray line represents the line of identity (y = x), indicating perfect agreement between predicted and ground truth TMTV. The solid red line represents the linear regression fit (R^2^ = 0.798, *p* < 0.001).

Representative examples of pTMTV and gtTMTV in the validation cohort are shown in Figure 3. In the validation cohort, the mean (±standard deviation) gtTMTV and pTMTV were 424.681 ± 498.811 cm^3^ (range: 3.584–2321.472 cm^3^) and 405.871 ± 466.984 cm^3^ (range: 7.616–2279.872 cm^3^), respectively. The mean absolute error (MAE) and mean absolute percentage error (MAPE) were 125.617 cm^3^ and 42.17%, respectively. The median absolute error (MedAE) and median absolute percentage error (MdAPE) were 41.600 cm^3^ and 32.34%, respectively. Bland–Altman analysis was performed to assess the agreement between gtTMTV and pTMTV (Figure 4). The results demonstrated excellent agreement, with deviations (±1.96 SD) of −18.81 (−529.44, 226.04 ± 1.96 SD), without significant bias (*p* > 0.05). Linear regression analysis showed a strong positive correlation between gtTMTV and pTMTV (R^2^: 0.798, *p* < 0.001) (Figure 4). The intraclass correlation coefficient (ICC) between gtTMTV and pTMTV was 0.892 (*p* < 0.001), indicating good agreement.

### 3.4. Predicted TMTV for Clinical Applications

**Figure 5:**
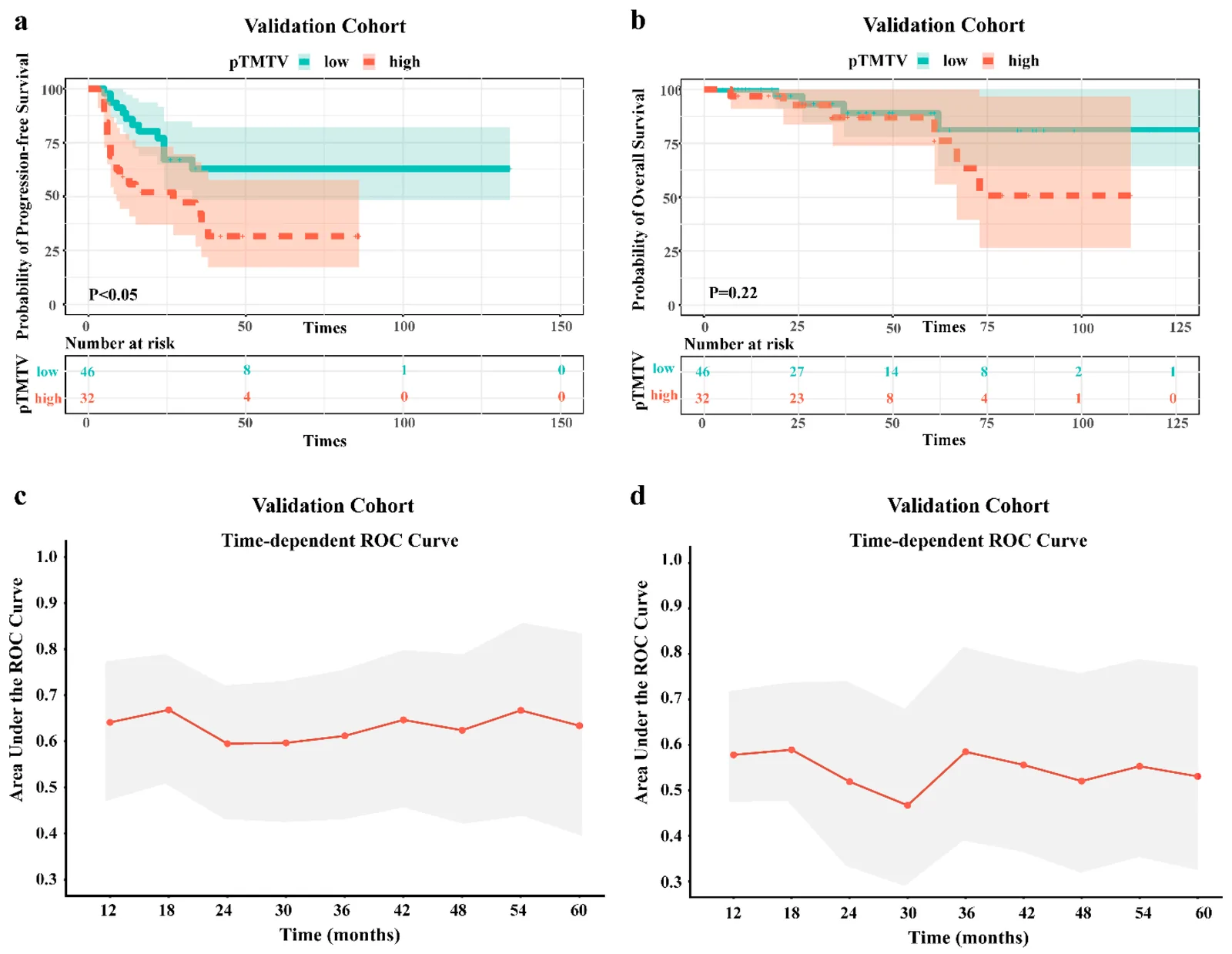
Survival of patients stratified by risk classification according to pTMTV for patients in the validation cohort for PFS (**a**) and OS (**b**); time-dependent area under the ROC curve of pTMTV for PFS (**c**) and OS (**d**) prediction in the validation cohort.

The cutoff value of pTMTV was determined as 323.781 cm^3^ based on the median value of the training cohort. Patients were subsequently stratified into high pTMTV and low pTMTV groups based on this threshold. KM analysis in the training cohort showed that patients in the high pTMTV group had a higher risk of adverse outcomes than those in the low pTMTV group (PFS: *χ*2: 4.1, *p* < 0.05; OS: *χ*2: 9.7, *p* < 0.05) In the independent external validation cohort, patients in the high pTMTV group had a higher risk of PFS events than those in the low pTMTV group (*χ*2: 7.7, *p* < 0.05). However, no significant difference in OS was observed between the two groups (*χ*2: 1.5, *p* = 0.22) (Figure 5). Furthermore, time-dependent ROC analysis was performed to assess the predictive performance of pTMTV at different time points. The results showed that pTMTV demonstrated strong predictive ability for PFS across multiple time points in the validation cohort. However, its predictive performance for OS was limited (Figure 5).

Cox proportional hazards regression was used to evaluate the prognostic value of pTMTV and clinical variables for PFS and OS. Univariate and multivariate analyses were performed to identify significant prognostic factors (Table 4). Univariate analysis showed that Ann Arbor stage, hemoglobin, and pTMTV were significant predictors of PFS [HR (95% CI): 1.961 (1.106–3.347), 1.958 (1.176–3.253), and 1.000 (1.000–1.001), respectively; all *p* < 0.05]. For OS, age and hemoglobin were also identified as significant prognostic factors [HR (95% CI): 2.596 (1.109–6.078) and 3.435 (1.386–8.516), respectively; both *p* < 0.05]. These variables were then included in the multivariate Cox regression analysis. Multivariate analysis showed that hemoglobin and pTMTV remained independent prognostic factors for PFS [HR (95% CI): 1.830 (1.093–3.062) and 1.000 (1.000–1.001), respectively; *p*: 0.021 and 0.048]. Additionally, age and hemoglobin were independent prognostic factors for OS [HR (95% CI): 2.369 (1.005–5.586) and 3.435 (1.386–8.516), respectively; *p*: 0.049 and 0.008].

## 4. Discussion

This study primarily used the MedNeXt deep learning model for segmenting PET images of HL patients. Tumor image segmentation currently mainly uses architectural models like CNN and U-Net, which are widely applied in PET image and PET/CT multimodal segmentation [5]. Rahimpour et al. [15] showed that multi-label CNN models perform well in glioma image segmentation. Wang et al. [16] found that the improved V-Net model based on U-Net could achieve relatively accurate lung cancer segmentation. Xu et al. [17] developed a U-Net-based deep learning framework that automatically detects and segments prostate cancer on PET/CT images, achieving favorable outcomes in small lesion segmentation. However, lymphoma mainly occurs in lymph nodes with widely distributed extranodal lesions and high variability, and requires differentiation from physiological uptake and drug clearance processes [13,18]. These factors make lymphoma segmentation more challenging than that of other tumors. MedNeXt shows significant differences in performance and applicability compared to other mainstream models. Unlike Transformer-based architectures like SwinUNETR [19], MedNeXt retains the inherent inductive bias of convolutional networks, making it easier to train on small-scale medical datasets with reduced overfitting risks. At the same time, it models long-range dependencies with large convolutional kernels, bypassing the Transformer’s need for extensive annotated data. Compared to traditional convolutional models like 3D U-Net [20], its inverted residual bottlenecks and compound scaling mechanisms greatly improve feature representation and model scalability. MedNeXt outperforms nnUNet [21] in segmentation accuracy, convergence speed, and cross-dataset generalization, especially showing more stable performance with multimodal data (e.g., CT, MRI) and datasets of varying scales. In the present study, we further benchmarked MedNeXt against five representative segmentation architectures (3D UXNet, nnFormer, nnU-Net, TransBTS, and UNETR) using the same data split, preprocessing pipeline, and evaluation framework. The results showed that MedNeXt achieved the best overall segmentation performance in the validation cohort, whereas nnU-Net delivered the most comparable results. These findings support the competitiveness of MedNeXt for HL PET segmentation in the current dataset. Nevertheless, further studies are warranted to determine whether segmentation models built on different backbone architectures differ in their downstream prognostic utility and clinical value.

During training, we set the patch size to 128 × 128 × 128. Although MedNeXt uses the nnU-Net pipeline for training, where the default patch size is typically based on the median voxel size of the dataset, studies [22] suggest that increasing it to 128 × 128 × 128 may improve performance. Cho et al. [22] achieved better results (mean DSC: 82.5, HD95: 3.5) in multimodal CT and MRI segmentation with a patch size of 128 × 128 × 128.

Segmentation models like MedNeXt typically optimize with a combination of Dice loss and cross-entropy (CE) loss. However, the standard CE loss with a weight of 1 often fails to penalize underrepresented classes adequately when dealing with class imbalance. To address this, we increased the CE loss weight to 1.7, thereby enhancing the model’s focus on pixels from minority classes. This adjustment improves recall while maintaining precision to some extent [23], reducing missed detections of small-class targets. Compared to standalone Dice loss, the CE loss provides smoother gradients [24], and integrating weighted CE stabilizes the training process, aiding gradient propagation in large-kernel ConvNeXt blocks. This strategy improves the model’s ability to identify tumor regions, ensuring robustness and reliability in real-world clinical applications.

In recent research on lymphoma image segmentation, Jiang et al. [12] used 3D U-Net for DLBCL segmentation, achieving a mean DSC of 0.78 ± 0.25. Tie et al. [25] developed the longitudinal-aware segmentation network (LAS-Net) for pediatric Hodgkin lymphoma PET image segmentation, reporting a Dice score of 0.77 (95% CI: 0.75–0.79). Yousefirizi et al. [18] proposed the TMTV-Net architecture, which uses a CNN-based two-step cascaded segmentation approach, attaining a DSC of 0.66 ± 0.16. In this study, PET images were used as input to train the MedNeXt model. The results showed that MedNeXt achieved a relatively high DSC (DSC: 0.702 ± 0.191), indicating good tumor segmentation accuracy and overlap, consistent with previous studies. However, the Hausdorff distance at the HD95 and ASSD showed relatively poor performance in the validation cohort. HD95 and ASSD are commonly used to quantify the maximum and average distances between the predicted and ground-truth tumor boundaries, with lower values indicating better boundary agreement. In this study, HD95 and ASSD were 67.9 ± 99.2 and 16.9 ± 31.659, respectively, indicating substantial boundary deviation between the predicted and ground-truth tumor regions, as well as limited spatial localization and contour delineation performance. This may be attributed to the limited ability of the model to detect and delineate small lesions. HL primarily involves the lymph nodes and lymphatic system, and lesions are typically small. Further analysis of outlier cases in the dataset revealed that patients with poor boundary delineation often had relatively low TMTV. Small lesions in the dataset may lead the segmentation algorithm to inadvertently include adjacent inflammatory tissues or regions with low metabolic activity, resulting in the overestimation of lesion boundaries and reduced tumor detection performance. Subsequently, TMTV was extracted based on the model-predicted tumor regions, and Bland–Altman analysis, ICC analysis, and linear regression analysis were performed. The results showed good agreement between gtTMTV and pTMTV in the validation cohort, consistent with previous studies [12,18,26].

TMTV is an independent risk factor for the prognosis of HL [3,4]. Some studies [12,27] have also shown that pTMTV, extracted using automatic segmentation, is prognostic for PFS and OS in DLBCL. However, a study by Leung et al. [28] reported that segmentation-derived metabolic parameters, such as MTV and TLA, were not associated with OSin prostate cancer. Currently, there are limited studies on the use of automatically segmentation-derived metabolic imaging parameters for predicting the prognosis of HL.

In this study, pTMTV was derived from automatically segmented tumor regions. Patients were stratified into high- and low-pTMTV groups using the median value from the training cohort. KM survival analysis was subsequently performed to evaluate survival outcomes. In the validation cohort, patients in the high-pTMTV group had a significantly higher risk of recurrence or progression than those in the low-pTMTV group (*p* < 0.05). However, no significant difference in OS was observed between the two groups. This may be because the assessment of overall survival (OS) was constrained by the limited number of clinical events (only 22 deaths across both cohorts, unevenly distributed). Consequently, there was insufficient statistical power to predict adverse OS outcomes. Future multicenter studies with larger sample sizes are warranted to further validate these findings. Time-dependent ROC analysis was also performed to assess the predictive performance of pTMTV. The results indicated that pTMTV demonstrated strong predictive ability for PFS at multiple time points in the validation cohort. However, its predictive performance for OS was limited. Cox proportional hazards regression analyses were subsequently performed. The results showed that pTMTV was an independent prognostic factor for PFS in patients with HL. However, it was not an independent predictor for OS, further indicating its limited predictive value for OS. TMTV reflects the overall metabolic activity of tumor lesions and provides a comprehensive assessment of tumor burden and disease extent. Therefore, a higher TMTV may serve as an indicator of poor prognosis and predict disease recurrence or progression. In this study, pTMTV was identified as an independent prognostic factor for PFS. It showed strong predictive performance at multiple time points and effectively distinguished patients with poor prognosis. However, its predictive ability for OS was limited. This discrepancy may be attributed to the imbalance in event distribution within the dataset. In addition to the limited number of positive events, the predictive value of pTMTV for OS may also be influenced by the survival benefit derived from second-line therapies initiated after disease progression in some patients [29]. We did not further investigate the prognostic value of pTMTV for OS specifically within this subgroup of patients who benefited from subsequent second-line treatment. Taken together with the limited and unevenly distributed number of events, these factors likely explain why pTMTV effectively stratifies PFS but fails to significantly distinguish OS.

The limitations of this study are as follows: First, this study is retrospective in nature and inherently carries a risk of selection bias. This may affect the reliability of the statistical analyses. Second, the sample size is limited, with few positive events and an imbalanced data distribution. Consequently, the statistical power is suboptimal. Therefore, prospective or multicenter studies are warranted to validate these findings. In addition, the MedNeXt architecture was trained solely on PET images, without integrating other imaging modalities such as CT or MRI. This limits the incorporation of complementary imaging information. Future research could explore multimodal fusion strategies to enhance model performance and improve the scientific rigor and innovation of the study. Finally, it is important to note that this study utilized conventional short-axial field-of-view (SAFOV) PET/CT systems. SAFOV acquisitions typically involve multiple bed positions, which may introduce motion artifacts and limit counting statistics, particularly in patients with extensive nodal and extranodal involvement. Recent advancements in long-axial field-of-view (LAFOV) PET/CT technology have demonstrated significant advantages, including improved signal-to-noise ratio (SNR), reduced radiation dose, and shorter acquisition times. Moreover, LAFOV systems have shown promise in refining small-lesion detection, mitigating boundary delineation errors, and enhancing the reproducibility of total metabolic tumor volume (TMTV) measurements [30,31]. Future studies incorporating LAFOV PET/CT may further optimize the accuracy of automated segmentation and prognostic stratification in lymphoma patients.

## 5. Conclusions

In this study, the MedNeXt model accurately segmented lymphoma lesions in PET images and enabled the extraction of TMTV. The pTMTV showed strong agreement with the reference standard. Furthermore, the pTMTV was identified as an potential independent prognostic factor for PFS. It facilitates risk stratification in patients with HL. However, the segmentation performance of the model requires further improvement. In addition, the predictive value of pTMTV for OS requires validation in studies with larger sample sizes.

## Figures and Tables

**Table 1:** Demographics and clinical characteristics of the study population.

Characteristic	Overall N = 192	Training Cohort N = 114	Validation Cohort N = 78	*p*-Value
Sex, n (%), Female/Male	114 (59%)/78 (41%)	67 (59%)/47 (41%)	47 (60%)/31 (40%)	0.837
Age, n (%), <45/≥45	120 (63%)/72 (38%)	74 (65%)/40 (35%)	46 (59%)/32 (41%)	0.404
LDH, n (%), Normal/Elevate	105 (55%)/87 (45%)	67 (59%)/47 (41%)	38 (49%)/40 (51%)	0.169
B system, n (%), No/Yes	123 (64%)/69 (36%)	72 (63%)/42 (37%)	51 (65%)/27 (35%)	0.752
Lymphocyte, n (%), Normal/Elevate	170 (89%)/22 (11%)	99 (87%)/15 (13%)	71 (91%)/7 (9.0%)	0.159
Lugano stage, n (%), I–II/III–IV	73 (38%)/119 (62%)	48 (42%)/66 (58%)	25 (32%)/53 (68%)	0.361
Extranodal involvement n (%), No/Yes	105 (55%)/87 (45%)	60 (53%)/54 (47%)	45 (58%)/33 (42%)	0.489
Hemoglobin, n (%), Normal/Elevate	113 (59%)/79 (41%)	70 (61%)/44 (39%)	43 (55%)/35 (45%)	0.385
Neutrophil, n (%), Normal/Elevate	105 (55%)/87 (45%)	56 (49%)/58 (51%)	49 (63%)/29 (37%)	0.061
WBC, n (%), Normal/Elevate	105 (55%)/87 (45%)	62 (54%)/52 (46%)	43 (55%)/35 (45%)	0.919
Albumin, n (%), Normal/Elevate	151 (79%)/41 (21%)	86 (75%)/28 (25%)	65 (83%)/13 (17%)	0.190
TMTV, Mean (SD)	436.31 (500.78)	444.26 (502.64)	424.68 (497.81)	0.288

Abbreviations: LDH, lactate dehydrogenase; WBC, white blood cell; TMTV, total metabolic tumor volume; differences were assessed by the χ2 test or Mann–Whitney U test.

**Table 2:** Summary of segmentation performance in the validation cohort.

	Validation Cohort			
	Mean	Median	IQR	SD
DSC	0.702	0.760	0.626–0.841	0.191
FNR	0.283	0.185	0.088–0.409	0.249
FPR	0.000258	0.000146	0.000049–0.000293	0.000358
HD95	67.998	29.231	8.000–82.156	99.220
Jaccard	0.561	0.615	0.456–0.726	0.203
NPV	0.9996	0.9998	0.9997–0.9999	0.0006
PPV	0.750	0.776	0.705–0.871	0.185
Sensitivity	0.716	0.814	0.590–0.911	0.249
SPE	0.9997	0.9998	0.9997–0.9999	0.0003
ASSD	16.992	6.627	3.184–14.679	31.659

Abbreviations: DSC, Dice similarity coefficient; FNR, false negative rate; FPR, false positive rate; NPV, negative predictive value; PPV, positive predictive value; SPE, specificity; HD95, 95th percentile Hausdorff distance; ASSD, average symmetric surface distance.

**Table 3:** Segmentation performance (mean ± SD) using different methods based on the validation cohort, with metrics including DSC, JAC, precision, and recall.

Method	DSC	Jaccard	PPV	Sensitivity
MedNeXt	0.702 ± 0.191	0.561 ± 0.203	0.750 ± 0.185	0.716 ± 0.249
3DUXNet	0.618 ± 0.236	0.487 ± 0.233	0.641 ± 0.253	0.690 ± 0.266
nnFormer	0.668 ± 0.228	0.538 ± 0.215	0.709 ± 0.216	0.705 ± 0.275
nnU-net	0.698 ± 0.199	0.567 ± 0.204	0.745 ± 0.192	0.721 ± 0.249
TransBTS	0.620 ± 0.247	0.491 ± 0.236	0.689 ± 0.246	0.670 ± 0.287
UNETR	0.485 ± 0.291	0.371 ± 0.264	0.512 ± 0.328	0.657 ± 0.276

**Table 4:** Univariate and multivariate analyses of factors predictive of PFS and OS.

Variables	Progression-Free Survival				Overall Survival			
	Univariate Analysis		Multivariate Analysis		Univariate Analysis		Multivariate Analysis	
	HR (95% CI)	*p* Value	HR (95% CI)	*p* Value	HR (95% CI)	*p* Value	HR (95% CI)	*p* Value
Sex, F/M	0.918 (0.545–1.544)	0.746	-	-	0.623 (0.254–1.529)	0.302	-	-
Age, <45/≥45	0.973 (0.576–1.646)	0.920	-	-	2.596 (1.109–6.078)	0.028	2.369 (1.005–5.586)	0.049
LDH level, normal/elevated	1.440 (0.864–2.400)	0.162	-	-	1.028 (0.443–2.384)	0.949	-	-
B symptoms, no/yes	0.790 (0.458–1.362)	0.396	-	-	1.735 (0.751–4.007)	0.197	-	-
Lymphocyte, normal/elevated	0.757 (0.326–1.762)	0.519	-	-	0.875 (0.203–3.780)	0.858	-	-
Ann Abor stage, I–II/III–IV	1.961 (1.106–3.347)	0.021	1.531 (0.822–2.852)	0.179	1.692 (0.662–4.325)	0.272	-	-
Extranodal involvement, no/yes	0.828 (0.497–1.380)	0.469	-	-	0.918 (0.396–2.127)	0.842	-	-
Hemoglobin, normal/elevated	1.958 (1.176–3.253)	0.010	1.830 (1.093–3.062)	0.021	3.659 (1.483–9.029)	0.005	3.435 (1.386–8.516)	0.008
Neutrophil, normal/elevated	1.249 (0.752–2.072)	0.390	-	-	1.297 (0.562–2.944)	0.542	-	-
WBC, normal/elevated	1.007 (0.605–1.675)	0.979	-	-	1.070 (0.462–2.478)	0.875	-	-
Albumin, normal/elevated	0.887 (0.471–1.669)	0.719	-	-	0.905 (0.304–2.691)	0.857	-	-
pTMTV	1.000 (1.000–1.001)	0.018	1.000 (1.000–1.001)	0.048	1.000 (1.000–1.001)	0.163	-	-

**Abbreviations:** F, female; M, male; LDH, lactate dehydrogenase; WBC, white blood cell; pTMTV, predicted total metabolic tumor volume.

## Data Availability

The datasets generated and analyzed during the current study are available from Nanjing Drum Tower Hospital, the Affiliated Hospital of Nanjing University Medical School, and Jiangsu Provincial People’s Hospital, and the First Affiliated Hospital of Nanjing Medical University.

## Abbreviations

The following abbreviations are used in this manuscript:

^18^F-FDG	^18^F-fluorodeoxyglucose
PET	Positron emission tomography
HL	Hodgkin lymphoma
TMTV	Total metabolic tumor volume
TLG	Total lesion glycolysis
SUV_max_	Maximum standardized uptake value
ROI	Region of interest
PFS	Progression-free survival
OS	Overall survival
HR	Hazard ratio
pTMTV	Predicted total metabolic tumor volume
gtTMTV	Ground truth total metabolic tumor volume
